# Two Measurement Set Partitioning Algorithms for the Extended Target Probability Hypothesis Density Filter

**DOI:** 10.3390/s19122665

**Published:** 2019-06-13

**Authors:** Yulan Han, Chongzhao Han

**Affiliations:** 1 School of Physics and Electronic-Electrical Engineering, Ningxia University, Yinchuan 750021, China; 2 School of Electronics and Information Engineering, Xi’an Jiaotong University, Xi’an 710049, China; czhan@mail.xjtu.edu.cn

**Keywords:** multiple extended target filter, partitioning algorithm, extended target tracking

## Abstract

The extended target probability hypothesis density (ET-PHD) filter cannot work well if the density of measurements varies from target to target, which is based on the measurement set partitioning algorithms employing the Mahalanobis distance between measurements. To tackle the problem, two measurement set partitioning approaches, the shared nearest neighbors similarity partitioning (SNNSP) and SNN density partitioning (SNNDP), are proposed in this paper. In SNNSP, the shared nearest neighbors (SNN) similarity, which incorporates the neighboring measurement information, is introduced to DP instead of the Mahalanobis distance between measurements. Furthermore, the SNNDP is developed by combining the DBSCAN algorithm with the SNN similarity together to enhance the reliability of partitions. Simulation results show that the ET-PHD filters based on the two proposed partitioning algorithms can achieve better tracking performance with less computation than the compared algorithms.

## 1. Introduction

In most multi-target tracking applications, it is assumed that each target produces one measurement per time step at most. This is reasonable for cases when the target’s extension is assumed to be negligible in comparison to sensor resolution. However, with the increase in sensor resolution capabilities, this assumption is no longer valid. In this case, different scattering centers of one target may give rise to several distinct detections varying from scan to scan, both in its number and the relative origin location. The correspoding cases can be found in the following situations: (1) Vehicles use radar sensors to track other road-users. (2) The ground radar stations track airplanes which are suffificiently close to the sensor. (3) Pedestrians are tracked using laser range sensors in mobile robotics. In addition, due to the high inner-density of the group target, it is neither practical nor necessary to track all individual targets within the target group. A group target can be tracked as a whole, and the problem formulation for tracking the group target is the same as that for tracking the extended target. Thus, in some works, such as [1,2,3,4,5,6,7], the extended target and the group target are studied together. Moreover, some studies take a group target as an extended target [1,4,5,6], which is also applied in this paper.

Extended target tracking has attracted much attention in the last decade. The studies on single extended target tracking mainly focus on measurement distribution, and the description and estimation of the target extension [1,6,7,8,9,10]. Multiple extended target tracking is a challenging problem due to the complexities caused by data association. In [11,12], the random matrix model and mixture RHMs were integrated into the probabilistic multi-hypothesis tracking (PMHT) framework to track multiple extended targets, respectively. In [13], the joint probabilistic data association (JPDA) was applied to tackle the problem of multiple extended target tracking. A box particle filter was developed to track multiple extended objects by combining interval-based techniques and the Bayesian framework [14]. Another way is based on a random finite set (RFS), such as the probability hypothesis density (PHD) filter [15], cardinalized probability hypothesis density (CPHD) filter [16] and multi-Bernoulli Filter [17]. In the work [18], an extended-target PHD (ET-PHD) filter was presented by extending the PHD filter to multiple extended targets. A CPHD filter for extended targets was proposed in [19], and a unified CPHD filter was proposed to track extended targets and unresolved group targets [20]. A Gaussian-mixture implementation of the ET-PHD filter was proposed in [21,22], called the extended target Gaussian-mixture PHD (ET-GMPHD) filter. The generalized labeled multi-Bernoulli filter was applied to track multiple extended targets based on the gamma Gaussian inverse Wishart model [17]. In [23], the Gaussian surface matrix was introduced into the PHD filter for multiple extended targets. Though those works based on RFS [17,18,19,20,21,22,23] can avoid explicit associations between measurements and targets, with all possible partitions of the measurement set need to be theoretically considered. In addition, the number of all possible partitions will grow dramatically with the increase in the number of measurements. Distance Partitioning (DP) and Distance Partitioning with Sub-Partitioning (DPSP) were proposed to obtain a reasonable subset of all possible partitions by Granstrom et al. in [22]. A fast partitioning algorithm based on a fuzzy ART model was proposed for the ET-GMPHD filter [24]. The algorithm consumed less computation time than DP without losing tracking performance. Since many of the cell and Gaussian mixture component pairs will be distant, the effect of updating that part of the PHD intensity with that cell will be negligible. According to this, Scheel et al. proposed a data segmentation method to alleviate computational complexity in [25]. The shape selection partitioning measurement partitioning algorithm was proposed in [26]. The algorithm first calculated potential centres and shapes of targets, and then combined each centre with different shapes to divide the measurements into subcells. In [27], the generalised distance partitioning (GDP), which applied the distance partitioning and Lmax-partitioning, was proposed to reduce the number of partitions and decrease computational complexity. The DBSCAN algorithm and the relaxed intersection were used to deal with data association and reduce the computational complexity in the data association process for a multiple extended target box particle filter [14].

When the density of measurements varies with the target, those measurement partitioning algorithms, which were applied Mahalanobis distance between measurements [28], cannot work well, such as DP, DPSP and GDP. In this paper, we employ the shared nearest neighbors (SNN) similarity [29], which can reflect the local configuration of measurements in the measurement space, to propose two measurement set partitioning approaches. The two approaches, SNN Similarity Partitioning (SNNSP) and SNN Density Partitioning (SNNDP), are relatively insensitive to variation in measurement density of extended targets. In SNNSP, the SNN similarity is introduced instead of the Mahalanobis distance between measurements, and the SNNDP is developed by combining the DBSCAN [30] with the SNN similarity to enhance the reliability of partitions.

Although it takes some time to calculate the SNN similarity, the ET-PHD filter based on the proposed partitioning approaches decreases the computational burden due to the less number of the resulting partitions. Especially in high clutter scenarios, a significant reduction in computational complexity can be achieved. Simulation results demonstrate that the proposed partitioning approaches can outperform the compared ones in several typical scenarios, namely, differing densities of measurements, high clutter and proximity among extended targets.

The rest of the paper is organized as follows. We briefly describe the problem formulation in Section 2. Section 3 provides a summary of the DP and DPSP, which are proposed in [22]. Section 4 presents two proposed partitioning approaches in this paper. In Section 5, simulation results are given to compare the performance of the ET-GMPHD filter using proposed partitioning algorithms with that using the compared algorithms. Section 6 presents concluding remarks and outlines future research directions.

## 2. Problem Formulation

The PHD measurement update equation for the extended target PHD filter, based on the Poisson multitarget measurement model [31], is derived in [18]. The corrected PHD-intensity, which is given by the multiplication of the predicted PHD-intensity and the measurement pseudo-likelihood, can be shown as

(1) $$\upsilon_{k|k}\left(x\right)=L_{Z_{k}}\left(x\right)\;\upsilon_{k|k-1}\left(x\right)$$

where $\upsilon_{k|k}$ is the corrected PHD-intensity; $\upsilon_{k|k-1}$ is the predicted PHD-intensity. $L_{Z_{k}}\left(x\right)$ is the measurement pseudo-likelihood function which can be defined as

(2) $$\begin{array}{cc} L_{Z_{k}}\left(x\right) & =1-\left(1-e^{-\gamma (x)}\right)\;p_{D}\left(x\right)+\\ & e^{-\gamma (x)}p_{D}\left(x\right)\sum_{p∠Z_{k}}\omega_{p}\sum_{W\in p}\frac{\gamma {\left(x\right)}^{\left|W\right|}}{d_{W}}\prod_{z\in W}\frac{\phi_{z}\left(x\right)}{\lambda_{k}c_{k}\left(z\right)} \end{array}$$

where, $p_{D}\left(\cdot \right)$ is the detected probability of the target; γ(·) is the mean number of measurements from one target; $\lambda_{k}$ is the mean number of clutter measurements per scan; $c_{k}\left(z\right)$ is the spatial distribution of the clutter over the surveillance region; $p∠Z_{k}$ means that the partition p partitions the measurement set $Z_{k}$ into non-empty cells *W*; W∈p denotes that the set *W* is a cell in the partition p; $\omega_{p}$ and $d_{W}$ are nonnegative coefficients defined for each partition and cell, respectively; $\phi_{z}\left(x\right)=f^{L}\left(z|x\right)$ is the likelihood function for a single target-generated measurement.

The first summation on the right hand side of (2) is taken over all partitions of the measurement set $Z_{k}$. All possible partitions of the measurement set need to be considered in theory. For example, the measurement set contains three individual measurements $Z_{k}=\left\{z_{k}^{1},z_{k}^{2},z_{k}^{3}\right\}$. All possible partitions of $Z_{k}$ are shown as follows

$$\begin{array}{cc} p_{1} & =\left\{\left\{z_{k}^{\left(1\right)},z_{k}^{\left(2\right)},z_{k}^{\left(3\right)}\right\}\right\}\;p_{2}=\left\{\left\{z_{k}^{\left(1\right)}\right\},\left\{z_{k}^{\left(2\right)}\right\},\left\{z_{k}^{\left(3\right)}\right\}\right\}\\ p_{3} & =\left\{\left\{z_{k}^{\left(1\right)},z_{k}^{\left(2\right)}\right\},\left\{z_{k}^{\left(3\right)}\right\}\right\}\;p_{4}=\left\{\left\{z_{k}^{\left(1\right)},z_{k}^{\left(3\right)}\right\},\left\{z_{k}^{\left(2\right)}\right\}\right\}\\ p_{5} & =\{\left\{z_{k}^{\left(1\right)}\right\},\left\{z_{k}^{\left(2\right)},z_{k}^{\left(3\right)}\right\}\} \end{array}$$

While the number of all possible partitions will grow dramatically with the increase in the number of sensor measurements. Thus, only considering a reasonable subset of all possible partitions is necessary to decrease computational complexity.

## 3. Review of Distance Partitioning and Distance Partitioning with Sub-Partitioning

### 3.1. Distance Partitioning (DP)

The DP introduced by Granstrom [22] is based on the distance between measurements. Given a set of measurements $Z={\left\{z_{i}\right\}}_{i=1}^{N_{z}}$ and a distance measure d(·,·), the distances between each pair of measurements can be calculated as

(3) $$\Delta_{ij}\;≜\;d\left(z_{i},z_{j}\right),\;\mathrm{for}\;1\leq i\neq j\leq N_{z}$$

Granstrom has proved that there is a unique partition that leaves all pairs (i,j) of measurements satisfying $\Delta_{ij}\leq d_{l}$ in the same cell. The algorithm is used to generate $N_{d}$ alternative partitions of the measurement set *Z*, by selecting $N_{d}$ different thresholds

(4) $${\left\{d_{l}\right\}}_{l=1}^{N_{d}},\;d_{l}<d_{l+1},\;\mathrm{for}\;l=1,\cdots ,N_{d}-1$$

The thresholds ${\left\{d_{l}\right\}}_{l=1}^{N_{d}}$ are selected from the set $D≜\left\{0\right\}\;\cup \;\left\{\Delta_{ij}\;|\;1\leq i<j\leq N_{z}\right\}$, and the Mahalanobis distance is selected as the distance measure d(·,·) in [22]. If one uses all of the elements in D to form alternative partitions, $\left|D\right|=N_{z}\left(N_{z}-1\right)/2+1$ partitions are obtained. In order to reduce the computational load, partitions are computed only for a subset of thresholds in the set D.

For two target-originated measurements $z_{i}$ and $z_{j}$ belonging to the same target, $d_{M}\left(z_{i},z_{j}\right)$ is $\chi^{2}$ distributed with degrees of freedom equaling to the dimension of the measurement vector. A unitless distance threshold can be computed as $\delta_{P_{G}}=\mathrm{invchi}2\left(P_{G}\right)$ for a given probability $P_{G}$, where invchi2(·) is the inverse cumulative $\chi^{2}$ distribution function. Granstrom et al. [21] have illustrated that good target tracking results are achieved with partitions computed using the subset of distance thresholds in D satisfying the condition $\delta_{P_{L}}<d_{l}<\delta_{P_{U}}$, for lower probabilities $P_{L}\leq 0.3$ and upper probabilities $P_{U}\geq 0.8$.

### 3.2. Distance Partitioning with Sub-Partitioning (DPSP)

The results given by the ET-GMPHD filter using the DP show the problem with underestimation of target set cardinality in the situations where two or more extended targets are spatially close [21]. In this situation, the DP will put those measurements from more than one target in the same cell *W*, and thus the ET-PHD filter based on the DP will interpret measurements from multiple targets as originating from the same target. The DPSP was proposed to remedy this problem in [22].

Suppose that a set of partitions using the DP has been obtained. Then, the maximum likelihood (ML) is applied to estimate the number of targets for each cell $W_{j}^{i}$, denoted by ${\hat{N}}_{x}^{j}$. If this estimate is larger than one, split the cell $W_{j}^{i}$ into ${\hat{N}}_{x}^{j}$ smaller cells, denoted by ${\left\{W_{s}^{+}\right\}}_{s=1}^{{\hat{N}}_{x}^{j}}$. Finally, a new partition, consisting of the new cells ${\left\{W_{s}^{+}\right\}}_{s=1}^{{\hat{N}}_{x}^{j}}$ along with the other cells in $p_{i}$, will be added to the list of partitions obtained by the DP. In [22], the K-means++ clustering, which modifies the standard K-means algorithm to overcome the problem that the cluster result is greatly affected by initial value [32], is applied to split measurements in the cell.

## 4. The Proposed SNN Partitioning

The partitions by the DP solely depend on the distances between each pair of measurements, ignoring the information from the local configuration of measurements. Though the distance thresholds are limited between $\delta_{P_{L}}$ and $\delta_{P_{H}}$, to reduce the computational load, the number of partitions still grows dramatically with the increase in the number of measurements. Moreover, a considerable number of partitions obtained by the DP are unreasonable, but will lead to high computational complexity of the ET-PHD filter.

**Figure 1:**
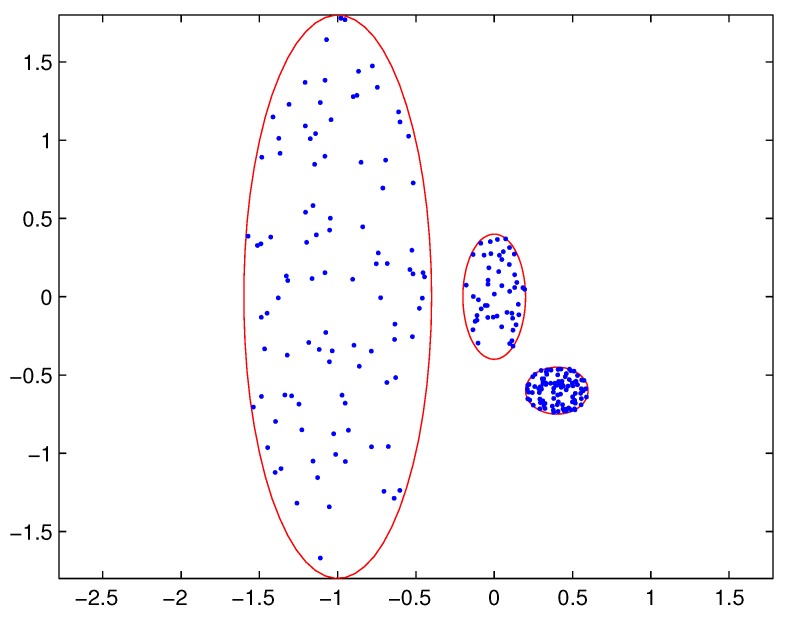
Measurements from three extended targets.

**Figure 2:**
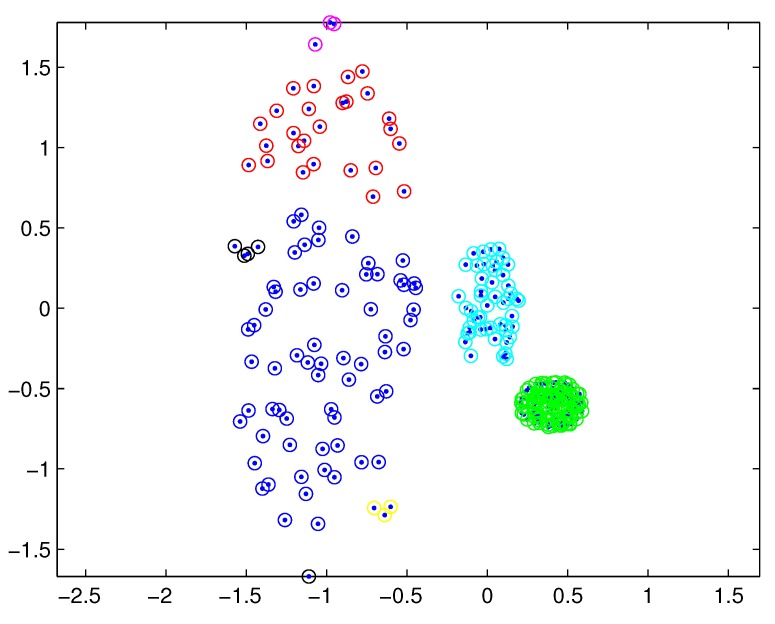
Partition by DP for a small threshold.

**Figure 3:**
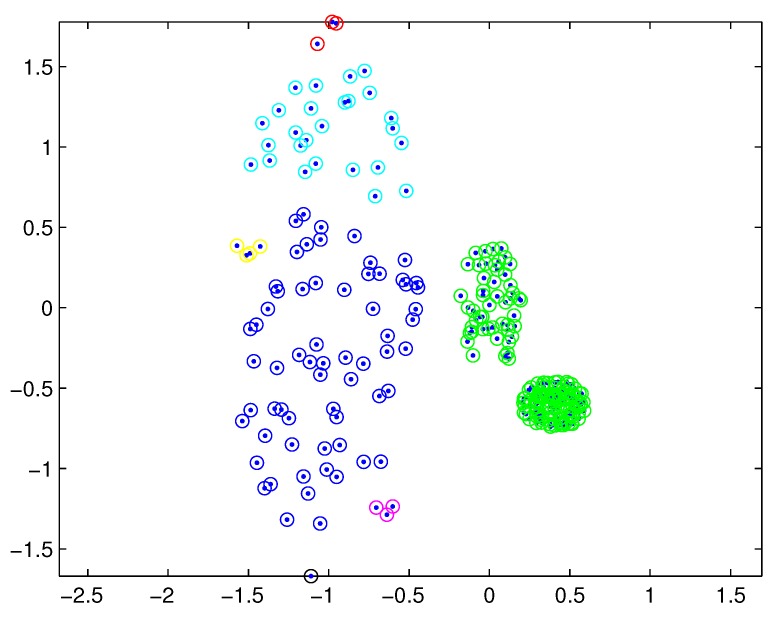
Partition by DP for a bigger threshold.

In practical applications, there may be a difference in the densities of measurement sources between different extended targets, and thus the density of measurements varies from target to target. In this case, reasonable partitions may not be included in the partitions by the DP. For example, Figure 1 depicts measurements from three extended targets with different measurement densities at a certain time scan. Measurements from the same extended target would be split into several small cells for a small threshold $d_{l}$, as shown in Figure 2 (one cell is represented by circles of the same color). On the other hand, for a slightly bigger threshold $d_{l+1}$, measurements from two targets are put in one cell as shown in Figure 3. Indeed, the appropriate distance threshold is unavailable for measurements shown in Figure 1, and the DP could not achieve the reasonable partition.

The computational complexity of the ET-PHD recursion is strongly dependent on the total number of cells in the resulting partitions. Since the clutter measurements are usually far away from each other, they tend to form individual multiple cells by the DP and DPSP. If there is a large amount of clutter measurements over the surveillance region, the number of cells constituted by clutter measurements is always far larger than that from targets. Thus, most computation of the ET-PHD filter based on the DP and DPSP is caused by dealing with the clutter.

For high clutter and the densities of measurements varing from target to target, we apply the SNN similarity, which reflects the local configuration of measurements in the measurement space to develop two measurement partitioning algorithms, SNNSP and SNNDP. The SNNSP only depends on the SNN similarity to decide whether two measurements are in the same cell or not. To promote the reliability further, we develop the SNNDP by combining the SNN similarity with the DBSCAN algorithm.

### 4.1. SNN Similarity

The SNN similarity was firstly proposed by Jarvis and Patric [29]. The SNN similarity between $z_{i}$ and $z_{j}$ is defined as the number of the nearest neighbors shared by the two measurements if and only if $z_{i}$ and $z_{j}$ have each other in their *K* nearest neighbor lists, as shown below

(5) $$S\left(z_{i},z_{j}\right)=\left|N\left(z_{i}\right)\cap N\left(z_{j}\right)\right|$$

Otherwise, the SNN similarity between $z_{i}$ and $z_{j}$ is zero. In (5), N(z) is the set of the *K* nearest neighbors of z, and $\left|A\right|$ means the cardinality of set *A*. The SNN similarity $S(z_{i},z_{j})$ is not larger than *K* according to (5).

### 4.2. SNN Partitioning Algorithm

This section introduces the SNNSP and SNNDP proposed in this paper.

#### 4.2.1. SNNSP

The SNNSP algorithm based on the SNN similarity puts all pairs of measurements satisfying $S\left(z_{i},z_{j}\right)\geq s_{l}$ in the same cell, and selects different similarity thresholds to obtain different partitions. The SNNSP can be described as the following steps.

- **Step 1**: Select the Mahalanobis distance as the distance measure d(·,·), and then compute the distance between each pair of measurements.
- **Step 2**: Find *K* nearest neighbors for each measurement, and then compute the SNN similarity between each pair of measurements.
- **Step 3**: Decide the similarity threshold set ${\left\{s_{l}\right\}}_{l=1}^{N_{s}}$. The similarity threshold set can be selected from its effective range, which is between the minimum and maximum of the SNN similarity, i.e., belonging to [1:K]. K is usually small, and so is the number of partitions by the SNNSP. Note that the neighborhood list size will decide the maximum of the upper similarity threshold.
- **Step 4**: For each given $s_{l}$, leave all pairs of measurements satisfying $S\left(z_{i},z_{j}\right)\geq s_{l}$ in the same cell. $N_{s}$ partitions of the measurement set *Z* can be generated by selecting $N_{s}$ different similarity thresholds. Some resulting partitions might be identical, and hence, need to be discarded so that each partition at the end is unique.

The SNN similarity reflects the local configuration of measurements in the measurement space, and has built-in automatic scaling. When measurements are widely spread, the volume containing *K* nearest neighborhoods expands, and conversely, the volume shrinks when measurements are tightly positioned. Therefore, the SNNSP does not critically depend on the distance thresholds, and is relatively insensitive to variations in density.

#### 4.2.2. SNNDP

To enhance the reliability of partitions further, we propose the SNNDP by combining the SNN similarity with the DBSCAN algorithm. In the DBSCAN algorithm [30], the density of a point is obtained by counting the number of points in a specified radius around the point. Points with a density above a specified threshold are classified as core points. A point considered as a border point is in the neighborhood region of a certain core point, while points which are neither core points nor border points are taken as noise points. Noise points are discarded, and the clusters are formed around the core points. If two core points are neighbors of each other, then their clusters are joined.

Let $NN_{i}$ denote the set of the measurements between which $z_{i}$ the SNN similarity is not less than a given threshold $s_{l}$. Then, the SNN density of $z_{i}$ can be regarded as the number of the meaurements in $NN_{i}$. Since measurements with high SNN densities tend to be generated from extended targets, they are considered as core measurements, and measurements with low SNN densities are taken as border measurements. The details of the SNNDP are as follows.

Steps 1–3 are the same as that in the SNNSP. For each given $s_{l}$, carry out step 4 and step 5 shown as below.

- **Step 4**: For a given $s_{l}$, compute the SNN density of every measurement. Measurements whose SNN densities are not less than a given SNN density threshold $U_{MP}$ are considered as core measurements, while those that are less than $U_{MP}$ but larger than 0 are considered as border measurements.
- **Step 5**: Leave all pairs of core measurements satisfying $S\left(z_{i},z_{j}\right)\geq s_{l}$ in the same cell. For the border measurement $z_{i}$, if the measurement $z_{j}$ is the nearest core point according to the SNN similarity, $z_{i}$ will be put in the cell where $z_{j}$ is.

The pseudo-code of the SNNDP is given in Table 1.

As done in the SNNSP, identical partitions must be discarded to ensure that each partition at the end is unique. It is noted that the SNNDP reduces to the SNNSP under the situation that $U_{MP}$ is 1.

The number of the partitions by the SNNSP and SNNDP is not larger than the neighborhood list size. Therefore, the computational complexity of the ET-PHD filter using the proposed partitioning algorithms is much less than that using distance partitioning, especially in the case of a large number of clutter measurements.

**Figure 4:**
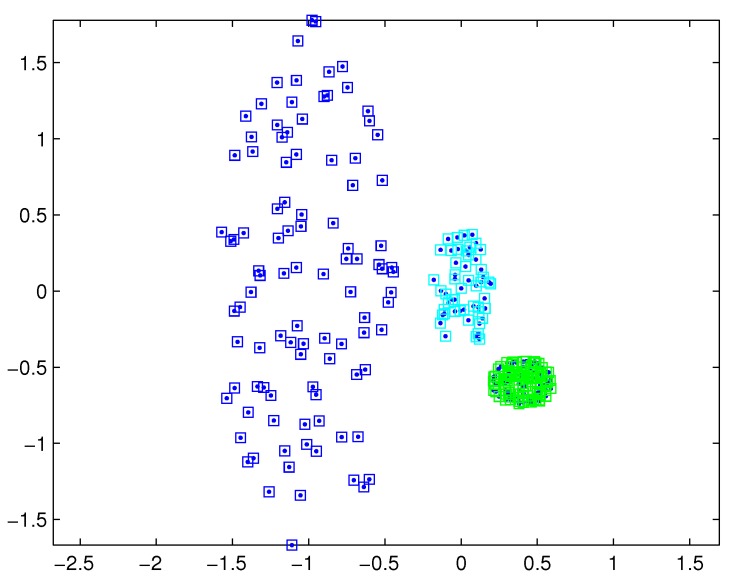
Partition by SNNSP for a certain similarity threshold.

**Figure 5:**
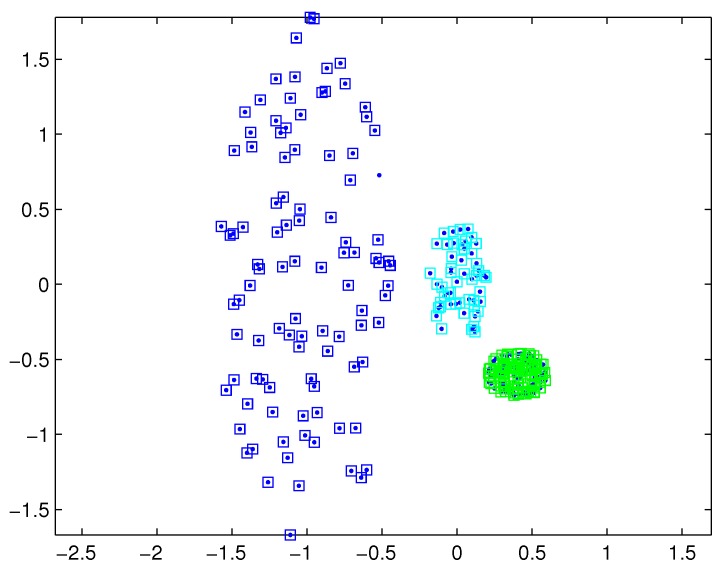
Partition by SNNDP for a certain similarity threshold.

The SNNDP (like the SNNSP) could also handle the situation that the densities of measurements varies from target to target. Reconsidering measurements shown in Figure 1, the SNNSP and SNNDP contain the basically correct partition as shown in Figure 4 and Figure 5, respectively, where the neighbor list size *K* is 20 and the SNN density threshold $U_{MP}$ is set to 5.

### 4.3. Parameterizations

The neighborhood list size *K* and the SNN density threshold $U_{MP}$ are two important parameters. If the neighborhood list size *K* is too small, the resulting partitions tend to focus on local variations, and thus, measurements from one extended target would be broken up into multiple cells even with the minimum similarity threshold. Conversely, if the neighborhood list size *K* is too large, the resulting partitions tend to neglect local variations, and thus, measurements from different extended targets would be put in one cell even with the maximum similarity threshold. The SNN density threshold $U_{MP}$ is usually set to be greater than 1 by the SNNDP, due to the SNNDP reducing to the SNNSP under the situation that $U_{MP}$ is 1. If the threshold SNN density $U_{MP}$ is too large, measurements from extended targets would be considered as clutter. $U_{MP}$ could be selected according to *K*, since the appropriate value of $U_{MP}$ mainly depends on *K* for given measurements and a given threshold $s_{l}$.

Simulation experiments are carried out to analyze the effect of *K* and $U_{MP}$ on the resulting partitions. The number of measurements is set to follow the Poisson distribution, and measurements from extended targets follow the uniform distribution and Gaussian distribution, respectively. Actually, there is no significant difference in the experimental results between Gaussian distribution and uniform distribution. Table 2 and Table 3 illustrate the range of *K* and $U_{MP}$, when the resulting partitions contain the correct partition or basically correct partition.

As can be seen from Table 2, the more the expected number of measurements per target is, the looser the requirement for the neighborhood list size *K* is. An appropriate value of the neighborhood list size *K* could be obtained even when there is a big difference in the expected numbers of measurements between different extended targets. From Table 3, it can be observed that the desirable range of $U_{MP}$ is large. In addition, the SNNSP and SNNDP wouldn’t work well if the expected number of measurements per target is small.

## 5. Simulation Results

In this section, simulation results are given to show the performance of the ET-PHD filter using the proposed partitioning approaches compared to using the compared algorithms. Here, we apply the Gaussian-mixture implementation of the ET-PHD filter (ET-GMPHD) proposed in [21,22]. Section 5.1 presents the simulation setup for tracking multiple extended targets. In Section 5.2, performance comparisons are conducted among the SNNSP, SNNDP, DP, DPSP and Distance Density Partitioning (DDP). The DDP applies the DBSCAN algorithm to cluster measurements and selects different specified radii to generate different partitions.

### 5.1. Simulation Setup

For illustration purposes, we consider a two-dimensional scenario over the surveillance region [−1000,1000]×[−1000,1000] (in m). The kinematic state of each extended target $x_{k}={\left[x_{k},y_{k},v_{k}^{x},v_{k}^{y}\right]}^{T}$ consists of the corresponding position components $\left(x_{k},y_{k}\right)$ and velocity components $\left(v_{k}^{x},v_{k}^{y}\right)$. ${\left[\cdot \right]}^{T}$ denotes the transpose of a matrix [·].

The dynamic evolution of each target state $x_{k}^{\left(i\right)}$ is assumed to follow a linear Gaussian model

(6) $$x_{k+1}^{\left(i\right)}=F_{k}\;x_{k}^{\left(i\right)}+G_{k}\;w_{k}^{\left(i\right)}$$

for $i=1,\ldots ,N_{x,k}$, where $F_{k}$ is the state transition matrix, $G_{k}$ is the noise gain, $w_{k}^{\left(i\right)}$ is Gaussian white noise with the covariance $Q_{k}^{\left(i\right)}$, and $N_{x,k}$ is the number of extended targets.

The sensor measurements from target *j* are generated according to the following linear Gaussian model

(7) $$z_{k}^{\left(j\right)}=H_{k}\;x_{k}^{\left(j\right)}+v_{k}^{\left(j\right)}$$

where $H_{k}$ is the observation matrix, and $v_{k}^{\left(j\right)}$ is white Gaussian noise with covariance $R_{k}^{\left(j\right)}$. Each target is assumed to give rise to measurements independently of the other targets.

The parameters in the dynamic and measurement models are shown as follows,

(8) $$\begin{array}{cc} F_{k} & =\left[\begin{array}{cccc} 1 & 0 & T & 0\\ 0 & 1 & 0 & T\\ 0 & 0 & 1 & 0\\ 0 & 0 & 0 & 1 \end{array}\right],\;G_{k}=\left[\begin{array}{cc} \frac{T^{2}}{2} & 0\\ 0 & \frac{T^{2}}{2}\\ T & 0\\ 0 & T \end{array}\right]\\ H_{k} & =\left[\begin{array}{cccc} 1 & 0 & 0 & 0\\ 0 & 1 & 0 & 0 \end{array}\right] \end{array}$$

with sampling time T=1 s, covariance matrices for process noise and measurement noise $Q_{k}=$ (2 m/s${}^{2}$) $\;I_{2}$ and $R_{k}=$ (20 m)${}^{2}\;I_{2}$, respectively. $I_{2}$ is 2×2 identity matrix.

The probability of survival is set to $p_{S}=0.99$, and the probability of detection is $p_{D}=0.99$. The birth intensity in the simulations is

(9) $$\upsilon_{b}\left(x\right)=0.1N\left(x;m_{b}^{1},P_{b}\right)+0.1N\left(x;m_{b}^{2},P_{b}\right)$$

with

(10) $$\begin{array}{cc} m_{b}^{1} & ={\left[250,250,0,0\right]}^{T} \end{array}$$

(11) $$\begin{array}{cc} m_{b}^{2} & ={\left[-250,250,0,0\right]}^{T} \end{array}$$

(12) $$\begin{array}{cc} P_{b} & =\mathrm{diag}([100,100,25,25]) \end{array}$$

The spawn intensity is

(13) $$\upsilon_{\beta }\left(x\right)=0.05N\left(x;\xi ,P_{\beta }\right)$$

where $P_{\beta }=\mathrm{diag}\left(\left[100,100,400,400\right]\right)$, and ξ is the target from which the new target is spawned.

### 5.2. Scenarios and Results

In this section, two scenarios are used to validate the performance of the SNNSP and SNNDP. The neighborhood list size *K* is set to 10. The density threshold $U_{MP}$ of the SNNDP and DDP is set to 3. To keep the number of Gaussian components at a computationally tractable level, the pruning and merging algorithm is performed as in [33]. After pruning and merging, the ET-GMPHD filter selects the means of the Gaussian components that have weights greater than some threshold, e.g., 0.5, as multiple extended target state estimates.

The OSPA distance makes a comprehensive evaluation for the estimated number of targets and estimates of kinematic states. It was considered as a metric for performance evaluation of the multitarget filter in [34,35] and is widely applied in recent several years [20,21,22,36]. However, the OSPA distance is not suitable for comparing the performance of the ET-PHD filter with different partitioning approaches. If the cut-off parameter of the OSPA distance is relatively small, the big error in the estimated kinematic states could not be reflected adequately. On the contrary, if the cut-off parameter is set to a big value, the OSPA distance stresses the error of the estimated number of targets, ignoring the estimation error of kinematic states.

In this paper, the performance of the multi-target filter on kinematic states is evaluated by the mean error based on an $L_{2}$—Wasserstein metric, which is defined as

(14) $$\begin{array}{c} E\left(X,\hat{X}\right)={\left(\frac{1}{M}\sum_{j=1}^{M}\left(\frac{1}{m}\min_{\pi \in \Pi_{n}}\;\sum_{i=1}^{m}d{\left(x_{i},{\hat{x}}_{\pi (i)}\right)}^{2}\right)\right)}^{1/2}\;\mathrm{if}\;m\leq n\\ E\left(X,\hat{X}\right)=E\left(\hat{X},X\right)\;\mathrm{if}\;m>n \end{array}$$

where, $X={\left\{x_{i}\right\}}_{i=1}^{m}$ is the set of kinematic states; $\hat{X}={\left\{{\hat{x}}_{i}\right\}}_{i=1}^{n}$ is the set of estimates of kinematic states; *M* is the number of Monte Carlo runs; d(·,·) is Euclidian distance; $\Pi_{n}$ represents the set of permutations of length *m* with elements taken from {1,2,⋯,n}. To analyse the computational complexity of the ET-GMPHD filter with different partitioning algorithms, the number of partitions, the number of cells, computational time of the partitioning algorithm, and computational time of the ET-GMPHD filter recursion per scan are given. They are obtained by averaging over 100 Monte Carlo runs, respectively.

#### 5.2.1. Differing Densities

**Figure 6:**
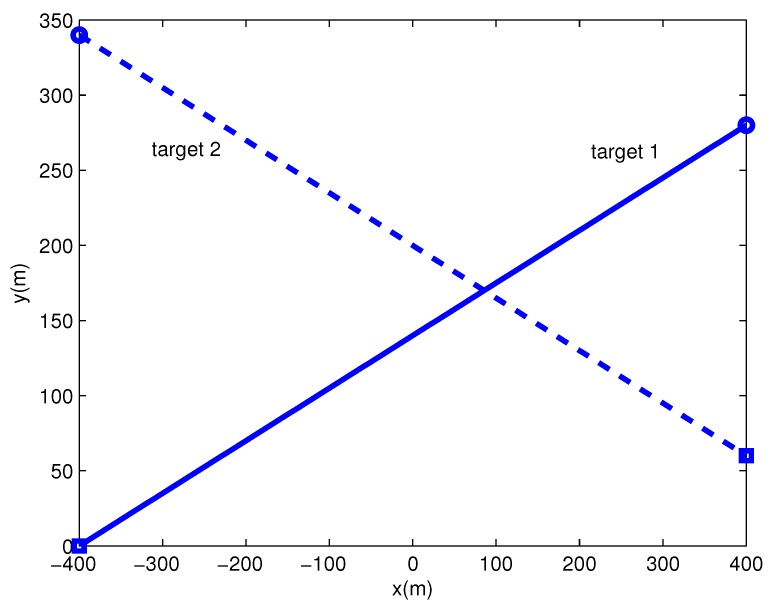
Trajectories of extended targets (’o’ is the start point, ${}^{\prime }{□}^{\prime }$ is the end point).

There are two extended targets with different measurement density in the surveillance region, and their trajectories are shown in Figure 6. Each extended target generates measurements per scan with Poisson rate 20. The density of measurements from target 2 is about five times that from target 1, and measurements from extended targets follow Gaussian distribution. Clutter measurements per scan are generated with Poisson rate 10, and uniformly distributed over the surveillance region.

**Figure 7:**
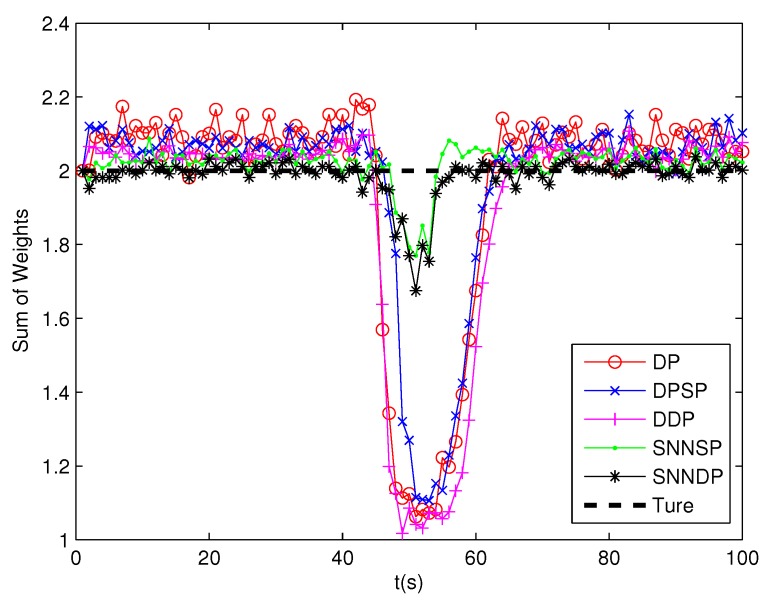
Sum of weights.

**Figure 8:**
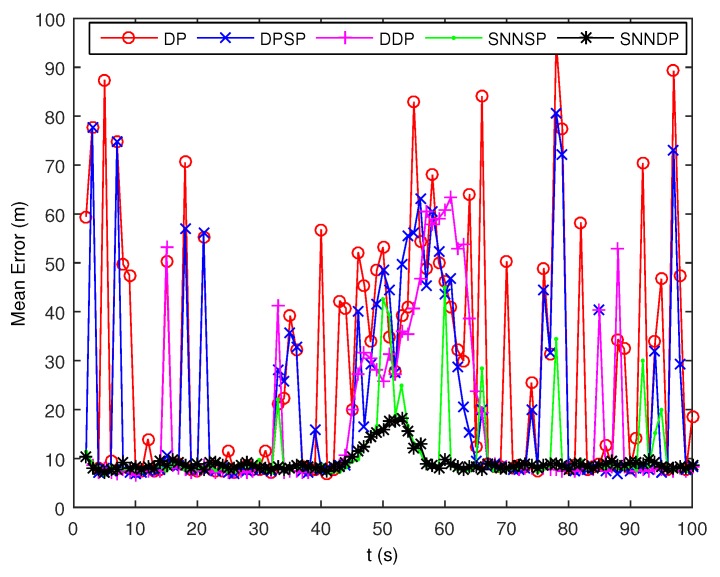
Mean error of estimated kinematic states.

As seen from Figure 7 and Figure 8, the DPSP only exhibits very slightly better than the the DP. The mean error of kinematic states estimated by the ET-GMPHD filter using the SNNSP and SNNDP is smaller than that using the DP, DPSP and DDP, and with a better estimate of the number of targets. The advantages are more evident when the two extended targets are spatially close at 45–60 s. The reason for this is that the SNN similarity measure reflects the local configuration of measurements in the measurement space, and thus is relatively insensitive to variations in density. In addition, due to the DDP and SNNDP incorporating the density information of the individual measurement, the ET-GMPHD filter with the DDP behaves better than that with the DP and DPSP, and the ET-GMPHD filter using the SNNDP outperforms that using the SNNSP, likewise.

**Figure 9:**
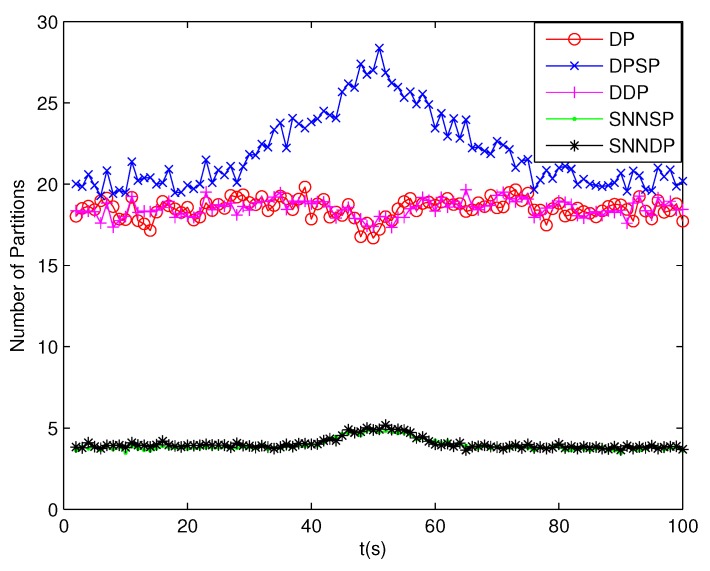
Number of partitions.

**Figure 10:**
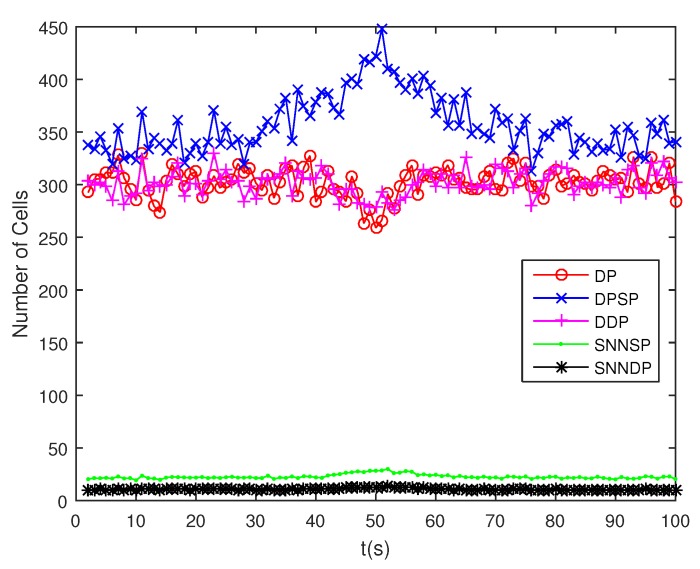
Number of cells.

**Figure 11:**
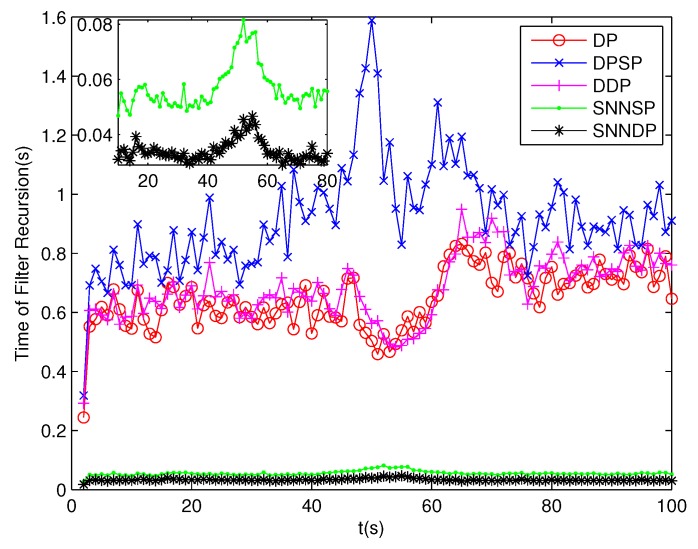
Computational time of the ET-GMPHD filter.

**Figure 12:**
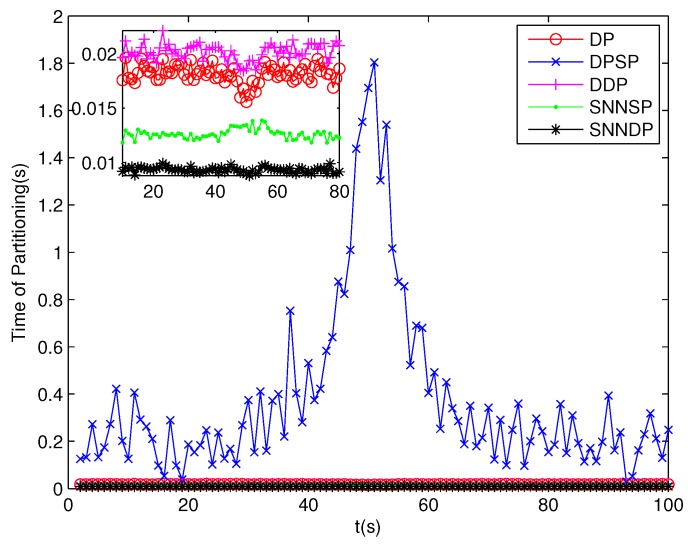
Computational time of Partitioning.

The number of partitions by the DP, DPSP and DDP grows rapidly with the increase in the number of measurements, while the number of partitions by the SNNSP and SNNDP mainly depends on the neighbor list size *K* which is a relatively small value. Therefore, as can be seen from Figure 9, the number of partitions by the SNNSP and SNNDP is smaller than that by the DP, DPSP and DDP. The number of cells is even less, about one-tenth, as shown in Figure 10. Updating one Gaussian component of the predicted PHD intensity by measurements in one cell, will form that of the posterior PHD intensity. Thus, the computational complexity of the ET-GMPHD recursion is strongly dependent on the number of cells in the resulting partitions. As can be seen from Figure 11, the computational time of the ET-GMPHD filter recursion using the SNNSP and SNNDP is dramatically smaller than that using DP and DDP. Although the SNNSP and SNNDP need to compute the SNN similarity for each pair of measurements, the computational time cost is usually less than that cost by DP and DDP due to the less number of their partitions, as shown in Figure 12. While the time required by DPSP is significantly longer because of adding partitions by Sub-Partitioning. Note that the simulations are implemented by Matlab2010 on Inter Core i5-4570 3.20 GHz processor and 4 GB RAM.

#### 5.2.2. High Clutter

In this scenario with high clutter, the trajectories of two extended targets are the same as in the above scenario (shown in Figure 6). Clutter measurements per scan are generated with Poisson rate 40 (four times the clutter level in the first scenario). Each extended target generates measurements per scan with Poisson rate 20, and the densities of measurements from two targets are set to be the same.

**Figure 13:**
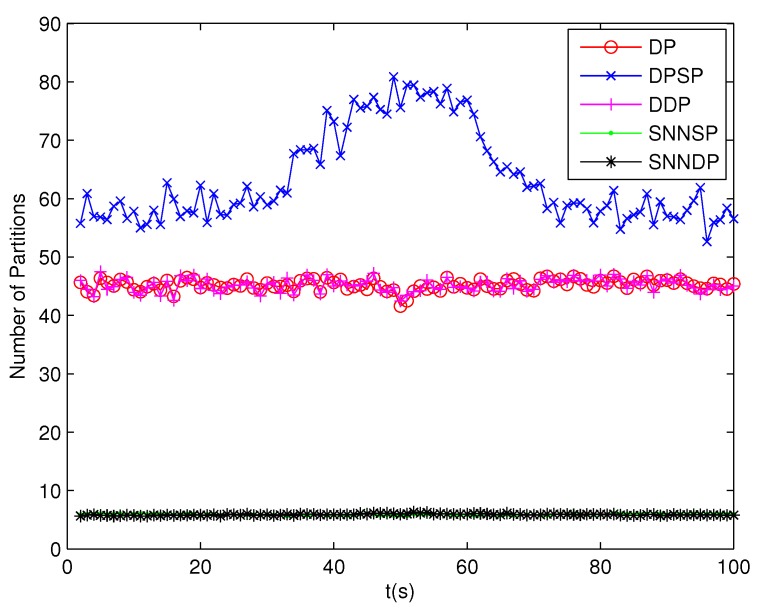
Number of partitions.

**Figure 14:**
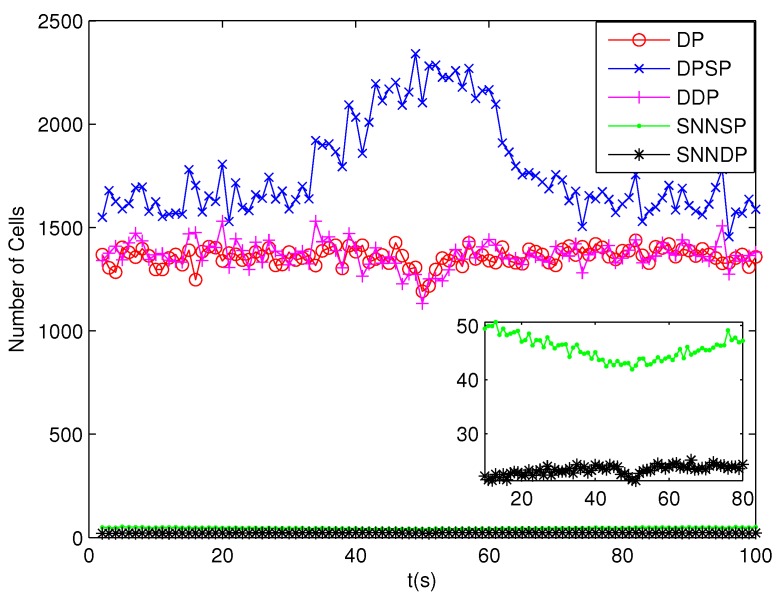
Number of cells.

**Figure 15:**
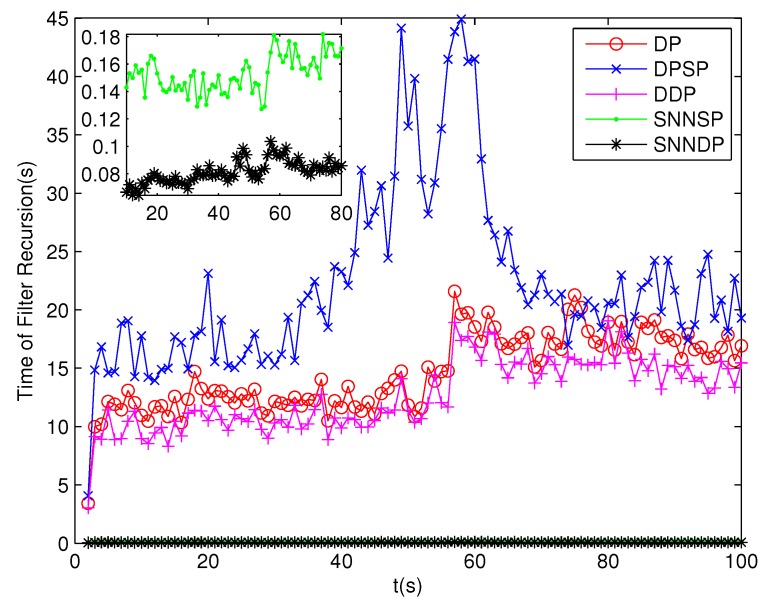
Computational time of the ET-GMPHD filter.

**Figure 16:**
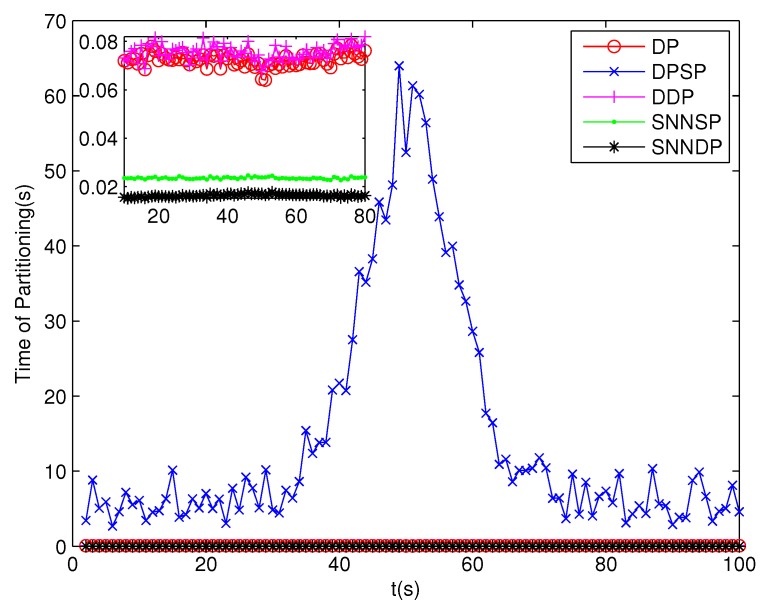
Computational time of Partitioning.

Because clutter measurements are distributed randomly in the surveillance region, the number of distance thresholds increases greatly in the presence of high clutter measurements. As can be seen from Figure 13 and Figure 14, the number of partitions and the number of cells obtained by the DP, DPSP and DDP are significantly larger, about ten and thirty times, respectively, than that by the SNNSP and SNNDP. As a result, the ET-GMPHD filters based on the SNNSP and SNNDP approaches indicate an evident decrease in computational time as shown in Figure 15, and the time of partitioning shows the same trend as shown in Figure 16.

**Figure 17:**
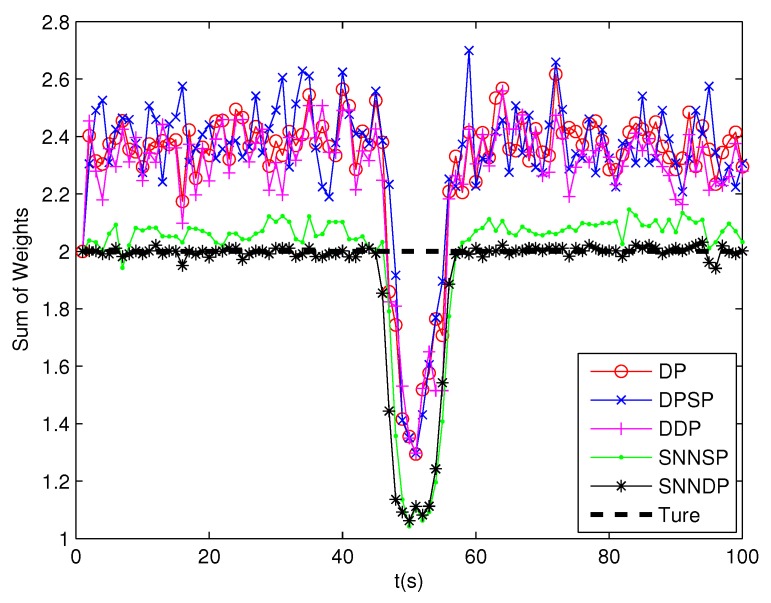
Sum of weights.

**Figure 18:**
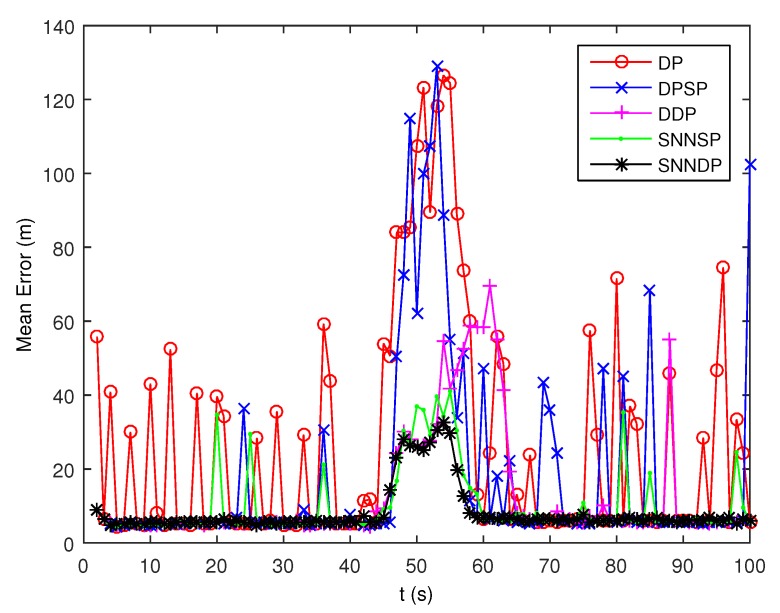
Mean error of estimated kinematic states.

The DP and DPSP tend to leave clutter measurements to individual multiple cells, and thus the extended target PHD filter may consider the clutter as a target in the high clutter environment. Consequently, the number of targets is overestimated when using DP and DPSP as shown in Figure 17. As shown from Figure 18, the mean error of the kinematic states given by the ET-GMPHD filter based on the proposed approaches is smaller than the one using DP, DPSP and DDP, especially in the situation that extended targets are spatially close.

## 6. Conclusions

This paper proposes two measurement set partitioning approaches for the ET-PHD filter based on the SNN similarity. The SNN similarity, which can reflect the local configuration of measurements in the measurement space, is applied to handle the situation that the density of measurements varies from target to target. To promote the reliability further, the SNNDP is developed by combining the SNN similarity with the DBSCAN algorithm. The resulting ET-PHD filter based on the proposed partitioning approaches decreases computational burden due to the smaller number of partitions. Especially in high clutter scenarios, a significant reduction in computational complexity can be achieved. Moreover, better estimates about the target number and the kinematic states can also be achieved in some challenging scenarios, such as differing densities of measurements, high clutter and proximity among extended targets. We will discuss the extension of the developed approaches for the extended target with complex shape, and extend our current result to the multi-sensor case with appropriate sensor management strategies [37,38] in our future work.

## Figures and Tables

**Table 1:** The pseudo-code of the SNNDP.

**Require:** $s_{l}$, $U_{MP}$, $S(z_{i},z_{j})$, $1\leq i\neq j\leq N_{z}$
Initialize: CoreBound(i) = 0, CellNumber(i) = 0, $1\leq i\leq N_{z}$ l=0
CellId = 1 the current cell id to 1
% Find the core measurements and boundary measurements
**for** $i=1:N_{z}$
num = 0
**for** $j=1:N_{z}$
**if** $S\left(z_{i},z_{j}\right)\geq s_{l}$
num = num + 1
**end if**
**end for**
**if** num $>U_{MP}$
CoreBound(i) = 1
**else if**
CoreBound(i) = −1
**end if**
**end for**
% Find cell numbers for core measurments
**for** $i=1:N_{z}$
**if** CellNumber(i) = 0 & CellBound(i) = 1
CellNumber(i) = CellId
CellNumbers = FindNeigbors(i,CellNumbers,CellId)
CellId = CellId + 1
**end if**
**end for**
% Find the cell of boundary measurements
**for** $i=1:N_{z}$
**if** CellNumber(i) = 0 & CellBound(i) = −1
**if** $S\left(z_{i},z_{j}\right)>S\left(z_{i},z_{m}\right)\left(m\neq j,1\leq m\leq N_{z}\right)$
CellNumber(i) = CellNumber(m)
**end if**
**end if**
**end for**
% the function FindNeigbors(·,·,·)
**function** CellNumbers = FindNeigbors(i,CellNumbers,CellId)
**for** $j=1:n_{z}$
**if** j≠i & $S\left(z_{i},z_{j}\right)\geq s_{l}$ & CellNumber(j) = 0 & CellBound(j) = 1
CellNumber(j) = CellId
CellNumbers = FindNeigbors(j,CellNumbers,CellId)
**end if**
**end for**

**Table 2:** Desirable range of the neighborhood list size.

Expected Number of Measurements per Target	Desirable Range of *K*
10	4–6
15	6–12
20	6–16
30	6–16
40	6–18
50	6–22
100	8–45

**Table 3:** Desirable range of the SNN density threshold.

Value of *K*	Desirable Range
*K*	of $U_{\mathrm{MP}}$
8	2–5
12	2–8
16	2–10
